# Effects of steam-assisted respiratory muscle training on sleep apnoea symptoms and pulmonary function in men and women: a pilot study

**DOI:** 10.1007/s11325-025-03449-2

**Published:** 2025-09-15

**Authors:** U. Al-Rammahi, T. Soukka, J. Malinen, RP Happonen, A. Sovijärvi, U. Anttalainen

**Affiliations:** 1 Division of Surgery and Cancer Diseases, Department of Oral and Maxillofacial Surgery, Turku University Hospital, Turku, Finland; 2 Sleep Research Center, Department of Pulmonary Diseases and Clinical Allergology, University of Turku, Turku, Finland; 3 Department of Mathematics and System Analysis, Aalto University, Espoo, Finland; 4 Department of Clinical Physiology, University of Helsinki, Helsinki, Finland; 5 Division of Medicine, Department of Pulmonary Diseases, Turku University Hospital, Turku, Finland

**Keywords:** Respiratory exercises, Sleep quality, Pulmonary function, Gender differences

## Abstract

**Purpose:**

Obstructive sleep apnoea (OSA) negatively impacts quality of life and increases cardiovascular and metabolic risks. Although continuous positive airway pressure is the gold-standard treatment, limited adherence reduces its clinical effectiveness. This study investigates whether steam-assisted respiratory muscle training (RMT) can alleviate symptoms, improve pulmonary function, and explore potential differences in response between men and women with OSA.

**Methods:**

This open-label, 12-week prospective pilot study included 60 participants with mild to moderate OSA, who performed individualized inspiratory and expiratory counter-pressure breathing exercises with steam inhalation. Pulmonary function tests, sleep-related questionnaires, and general health assessments were conducted at baseline and post-intervention. Participants showing notable improvements from baseline were classified as high responders, enabling subgroup analyses.

**Results:**

Final results of 33 of the 60 participants showed significant mean improvements, with the Insomnia Severity Index (ISI) decreasing by 1.8 points, the Pittsburgh Sleep Quality Index (PSQI) by 2 points, and the 12-item General Health Questionnaire (GHQ-12) by 7.9 points (all *p* < 0.01). Forced expiratory volume in one second (FEV₁) increased from from 3.6 L to 3.8 L (*p* = 0.04). Subgroup analysis revealed an enhancement in sleep-related symptoms and pulmonary function.

**Conclusions:**

RMT may serve as a patient-centered alternative for managing the symptomatic burden of mild to moderate OSA. While larger trials are needed to confirm these preliminary findings, these pilot results do not yet demonstrate sustained benefit and should be interpreted with caution.

Trial registration ClinicalTrials.gov, register no. NCT05320952.

**Supplementary Information:**

The online version contains supplementary material available at 10.1007/s11325-025-03449-2.

## Introduction

Obstructive sleep apnoea (OSA) is a common condition in which partial or complete obstruction of the upper airway disrupts respiration and normal sleep. This leads to frequent awakenings, impaired daytime function, and increased cardiovascular and metabolic risks [1, 2]. Although lifestyle interventions, surgical procedures, and mandibular advancement devices are possible treatments, continuous positive airway pressure (CPAP) is widely accepted as the gold-standard [3]. However, discomfort associated with CPAP often results in adherence rates under 40% [4, 5].

Growing evidence suggests that non-anatomical characteristics, such as upper airway collapsibility, arousal threshold, and respiratory control stability, also contribute to OSA pathophysiology [6, 7]. This understanding has opened the door for therapies targeting these mechanisms. In this context, respiratory muscle training (RMT) has shown promise in improving airway stability and oxygenation in patients with sleep apnoea. However, previous studies have often used devices with varying mechanisms and have been conducted in predominantly male populations, leaving questions about optimal training protocols and potential gender-specific responses unanswered [8, 9].

WellO2^®^ device integrates inspiratory and expiratory resistance exercises with heated steam. Previous work in other respiratory conditions, such as asthma, supports its capacity to enhance respiratory muscle performance and to improve quality of life [10]. While RMT with the WellO2^®^ device has not been previously studied in OSA, this approach is compelling; the combination of resistance training may strengthen pharyngeal muscles, while the humidified air may soothe and hydrate upper airway mucosa, potentially reducing collapsibility. This differs from prior RMT studies that focused solely on inspiratory or expiratory training without steam [11, 12].

The present clinical intervention pilot study aimed to determine whether 12 weeks of steam-assisted RMT could alleviate symptoms, improve respiratory function, and explore potential gender-based differences in outcomes in adults with mild to moderate OSA. The focus on patient-centered, self-administered approaches addresses a growing clinical need for alternatives or add-on therapies to standard CPAP, especially for those facing comfort and adherence challenges.

## Methods

### Study design and participants

A pilot study with prospective clinical intervention study enrolled 60 employed adults aged 18–60 years with mild to moderate OSA (apnoea-hypopnoea index [AHI] of 5–29/h) were recruited from the Pulmonary Clinic at Turku University Hospital between May 2022 and February 2024. All participants diagnoses were confirmed by home cardiorespiratory polygraphy in primary care clinics before referral to the Pulmonary clinic for treatment. Final inclusion into the study was based on the first overnight polysomnography (PSG) demonstrating an AHI of less than 30 per hour. Other exclusion criteria were ongoing sleep apnoea treatments, major oral or airway surgery, body mass index (BMI) > 40 kg/m², severe other pulmonary diseases (chronic obstructive pulmonary disease, asthma, pulmonary fibrosis, lung cancer), severe heart failure (NYHA 3–4), history of stroke with permanent symptoms or neuromuscular disease, unemployment, pregnancy, and disability to give the written informed consent to the study.

The study protocol was approved by the Clinical Research Centre of the Turku University Hospital (T37/2021) and registered in ClinicalTrials.gov (NCT05320952). Ethical approval was granted by the Southwest Finland Hospital District Ethics Committee (ETMK 31/1801/2021). The Finnish Medicines Agency (FIMEA) authorized the use of the WellO2^®^ device (WellO2 Oy, Finland).

### Intervention

Participants completed a 12-week upper-airway respiratory muscle training program. Training consisted of inspiratory and expiratory counter-pressure breathing performed through a mouthpiece using a dedicated device that delivered adjustable steam and stepless adjustable resistance (settings 0-3, corresponding approximately to 15, 30, 65, and 120 cmH₂O, respectively). The device (WellO₂, WellO₂ Oy, Finland) combines exhalation into a heated water reservoir with inhalation through the same circuit, producing warm, humidified airflow and counter-pressure.

At baseline, maximal inspiratory and expiratory pressures (MIP/MEP) were measured with a MicroRPM^®^ device (CareFusion, UK). The target counter-pressure for each phase (inspiration or expiration) was then individually set to 30% of the measured MIP or MEP, respectively. Steam temperature in the training device was individually set to 55, 60, or 65 °C based on participant comfort.

Participants performed two sessions per day, each lasting up to 15 min, throughout the 12-week period. Sessions consisted of three sets of five deep, slow breathing against the device-generated counter-pressure via the mouthpiece, with a short rest between sets. The resistance setting (0-3) could be adjusted steplessly on the device to achieve the prescribed workload while maintaining a comfortable breathing pattern.

Proper technique and device operation were taught at initiation. Adherence support was provided via a mid-study follow-up call. Participants recorded use in paper diaries during three separate 2-week windows (beginning, middle, and end of the intervention), including session dates/times and device settings (temperature and resistance). Diaries were reviewed to assess adherence and protocol fidelity.

Usual care continued unchanged unless clinically indicated. Participants were instructed to stop a session if they experienced significant discomfort and to contact the study team; no other protocolized safety interventions were required. Device hygiene and handling followed the manufacturer’s general instructions.

### Measurements and assessments

Baseline evaluations included demographic data (age, gender, smoking status), anthropometric measurements (height, weight, BMI, waist and neck circumference), and medical history including medications. PSG established OSA severity at baseline and post-intervention. Pulmonary function tests with a MicroLab^®^ spirometer (CareFusion, UK) were performed to measure forced expiratory volume in one second (FEV₁), forced vital capacity (FVC), and peak expiratory flow (PEF). MIP and MEP were recorded to gauge muscle strength and measured by using a MicroRPM^®^ device (CareFusion, UK). Participants performed repeated inspiratory and expiratory maneuvers from residual volume (for MIP) or total lung capacity (for MEP) following established guidelines from the European Respiratory Society (ERS) and American Thoracic Society (ATS) [13]. The highest pressure (cmH₂O) sustained for at least one second was recorded for each maneuver. Participants were instructed to perform at least three acceptable maneuvers [14].

Subjective outcomes were evaluated using validated questionnaires in their Finnish versions. The Epworth Sleepiness Scale (ESS) assessed daytime sleepiness, with a maximum score of 24; scores ≥ 10 indicate excessive daytime sleepiness [15], the Pittsburgh Sleep Quality Index (PSQI) measured sleep quality, with a maximum score of 21; scores > 5 indicate poor sleep quality [16], its components include subjective sleep quality, sleep latency, sleep duration, habitual sleep efficiency, sleep disturbances, use of sleeping medication, and daytime dysfunction; the Insomnia Severity Index (ISI) evaluated sleep disturbance, with a maximum score of 28; scores ≥ 15 indicate clinical insomnia [17, 18] and the Depression Scale (DEPS) screened for depressive symptoms, with a maximum score of 30; scores ≥ 9 are considered indicative of depression [19]. General mental health was measured by the 12-item General Health Questionnaire (GHQ-12), with a maximum score of 36; scores ≥ 12 suggest potential psychological distress [20]. In addition, a clinical sleep apnoea symptom questionnaire was used to assess common symptoms of obstructive sleep apnea, including daytime sleepiness, morning headaches, snoring, nocturnal awakenings, and sleep-related breathing disturbances with three possibilities to answer the frequence of symptoms: often (scored as 3), sometimes (scored as 2) or never (scored as 1). Responses were collected at baseline and post-intervention.

### Statistical methods

Data was analyzed using IBM SPSS Statistics (version 29.0.0.0). Normality was assessed with the Shapiro-Wilk test. Depending on data distribution, paired t-tests or Wilcoxon signed-rank tests were used to compare pre- and post-intervention outcomes. Welch’s t-test was applied for between-group comparisons with unequal variances. The participants showing individual improvement from baseline were classified as high responders, enabling subgroup analyses.

Linear regression identified continuous predictors of outcome changes. Binary logistic regression was used to examine whether baseline characteristics, including central obesity and multimorbidity, predicted clinically significant symptom thresholds. Outcomes were dichotomized using literature-based thresholds: ISI ≥ 15, ESS ≥ 10, DEPS ≥ 9, GHQ-12 ≥ 24, and PSQI ≥ 5. Central obesity was defined gender-specifically (waist ≥ 102 cm for men, ≥ 88 cm for women) according to WHO and NCEP-ATP III guidelines [21]. Rare comorbidities were collapsed into a binary “Any Comorbidity” variable to preserve statistical power.

To minimize overfitting, all logistic models adhered to the ≥ 5 events-per-variable (EPV) rule [22]. Bonferroni correction for multiple comparisons was applied, both unadjusted and Bonferroni-adjusted p-values are reported. Statistical significance after correction was set at *p* < 0.05.

## Results

### Study cohort and characteristics

**Fig. 1:**
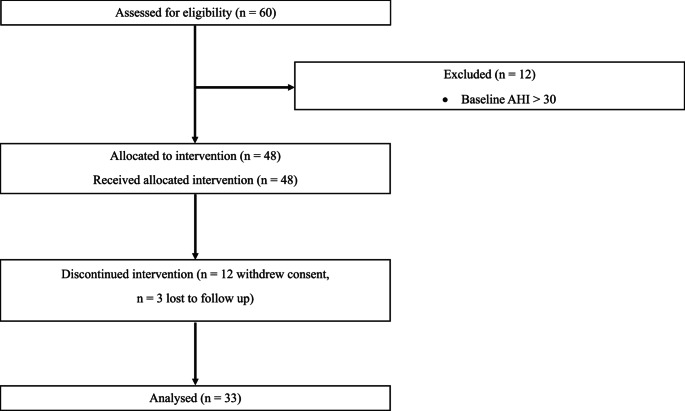
CONSORT diagram illustrating participant enrollment, allocation, and follow-up

Thirty-three participants (17 men, 16 women) out of 60 completed the 12-week study (Fig. 1).

Some differences were observed between those who completed the study and those who were excluded. Excluded participants had a significantly higher mean BMI, higher PSQI scores and lower pulmonary values. Compared to men, the included women had significantly smaller neck circumference, and lower MEP and pulmonary function values including PEF, FVC, FEV₁, and FEV₁% (Table 1).

**Table 1:** Included and excluded participants’ characteristics at baseline

	Included Participants		Participants		
Variable	Men	Women	Included	Excluded	*p*
Sample	17 (55.8%)	16 (44.2%)	33 (55%)	27 (45%)	0.4
Age	46 ± 8.5	50 ± 9.5	47.9 ± 9.1	46 ± 9.2	0.4
BMI (kg/m²)	28.3 ± 4.3	30.5 ± 4.7	28.6 ± 5.2	31 ± 2.9	**0.04**
Smoking (pack-years)	24 ± 17.8 (*n* = 5)	55.5 (*n* = 1)	29.3 ± 20.5 (*n* = 6)	18.6 ± 13.1 (*n* = 7)	0.3
Waist (cm)	103.1 ± 12	101.5 ± 15	102.3 ± 13.6	103 ± 11.4	0.8
Neck (cm)	42.2 ± 3*	37.9 ± 3*	38.9 ± 4.4	38.1 ± 2.5	0.1
AHI (/h)	18.9 ± 6.4	20.5 ± 6.2	19.6 ± 6.3	30.1 ± 19.5	**0.01**
MIP (cmH₂O)	69.4 ± 24.1*	54 ± 25.6*	64.9 ± 22	54.8 ± 19.5	0.07
MEP (cmH₂O)	79.4 ± 35*	55.4 ± 22.3*	72 ± 30	54.5 ± 20.7	**0.04**
PEF (L/min)	621.4 ± 114 *	448.3 ± 61*	538.1 ± 127	466.3 ± 67	**0.007**
FVC (L)	5.05 ± 0.63*	3.08 ± 0.65*	4.1 ± 1.2	3.7 ± 1.2	0.2
FVC (% of predicted)	102.6 ± 11.1*	88 ± 21.8*	101.1 ± 13.1	92.4 ± 21.3	0.07
FEV₁ (L)	4.3 ± 0.53*	2.79 ± 0.49*	3.6 ± 0.6	3.3 ± 0.9	0.2
FEV₁ (% of predicted)	109.5 ± 12.5*	92.8 ± 17.2*	98.6 ± 16.6	98.5 ± 20.3	1
ESS	5.7 ± 3.6	8.4 ± 5.3	6.9 ± 4.5	6.3 ± 3.2	0.5
DEPS	4.4 ± 3.4	4.9 ± 4.4	4.6 ± 3.8	3.9 ± 2.9	0.4
GHQ-12	23.2 ± 5.3	22.9 ± 5.6	23.1 ± 5.3	22.8 ± 4.6	0.8
PSQI	11.3 ± 3.4	13.1 ± 5.5	12.1 ± 4.5	15 ± 4.1	**0.01**
ISI	9.3 ± 4.9	8.9 ± 4.4	9.2 ± 4.6	8.3 ± 5	0.5

Values are presented as mean ± standard deviation. Asterisk (*) indicates a statistically significant difference (*p* < 0.05) between included men and women, according to Welch’s T-test. P-values presented in the last column represent comparisons between included and excluded participants, according to Welch’s T-test. Abbreviations: BMI = Body Mass Index. AHI = apnea-hypoapnea index, MIP = Maximal Inspiratory Pressure, MEP = Maximal Expiratory Pressure, PEF = Peak Expiratory Flow, FVC = Forced Vital Capacity, FEV₁ = Forced Expiratory Volume in one second. ESS = Epworth Sleepiness Scale, ISI = Insomnia Severity Index, PSQI = Pittsburgh Sleep Quality Index, DEPS = Depression Scale, GHQ-12 = 12-item General Health Questionnaire

Comorbidities were present in 76% (*n* = 25) of included participants, mainly cardiovascular and metabolic disorders. Of these, 12 (48%) had one or two and 14 (52%) had three or more comorbidities. Hypertension was most common (*n* = 8), followed by cerebrovascular disease (*n* = 6) and benign prostatic hyperplasia (*n* = 5). The prevalence of comorbidities of the included participants did not differ significantly between genders. The total list of comorbidities is shown in Supplement 1.

Participants’ regular medications were classified according to the Anatomical Therapeutic Chemical classification system. Lipid-lowering agents were the most used (*n* = 9), followed by antidepressants (*n* = 8), and ACE inhibitors or angiotensin receptor blockers (*n* = 7) for hypertension. Hormonal contraceptives were used by six participants, and five participants used acid-suppressing medications. Medication usage of the included participants did not differ significantly between genders. All medications are listed in Supplement 2.

### Symptom outcomes

As summarized in Table 2, the overall study group experienced significant reductions post-intervention in insomnia severity (ISI), general mental distress (GHQ 12), and sleep disturbance component (PSQI: Sleep disturbance: baseline 1.2 ± 0.4, post-intervention 1.1 ± 0.2, mean difference 0.1, *p* = 0.02), whereas daytime sleepiness (ESS) and depression scores (DEPS) or other components of PSQI showed no consistent change. A subgroup of participants demonstrated more pronounced improvements in all validated questionnaires (Table 3). The prevalence of symptoms did not differ significantly between genders. A detailed analysis of the PSQI component scores was conducted, revealing a statistically significant improvement only in the sleep disturbance component (*p* = 0.02) (data not shown).

**Table 2:** Changes in symptom parameters in participants following respiratory muscle training

Symptom Measure	Baseline			Post-Intervention			Mean Difference (95% CI)			Total		
	Men	Women	Total	Men	Women	Total	Men	Women	Total	Hedges’ g	*p*	adj *p*
ESS	5.7 ± 3.6	8.4 ± 5.3	6.9 ± 4.5	5.5 ± 4.2	7.4 ± 4.8	6.3 ± 4.5	−0.2 (−0.5–0.9)	−1 (−0.6–2.6)	−0.6 (−0.2–1.3)	−0.3	0.1	0.5
ISI	9.3 ± 4.9	8.9 ± 4.4	9.2 ± 4.6	7.5 ± 5.1	7.3 ± 5.6	7.4 ± 5.2	−1.8 (−0.2–3.8)	−1.6 (−0.02–3.3)	−1.8 (0.5–3)	−0.5	**0.009**	**0.05**
DEPS	4.4 ± 3.4	4.9 ± 4.4	4.6 ± 3.8	4.5 ± 4.4	4.3 ± 4.9	4.4 ± 4.6	0.1 (−1.2–0.9)	−0.6 (−0.1–1.4)	−0.2 (−0.5–0.9)	−0.1	0.6	1
GHQ-12	23.2 ± 5.3	22.9 ± 5.6	23.1 ± 5.3	14.9 ± 3.4	15.6 ± 10.1	15.2 ± 8.1	−8.3 (4.4–12.1)	−7.3 (2.9–11.8)	−7.9 (5.1–10.6)	−1	**< 0.001**	**< 0.005**
PSQI	11.3 ± 3.4	13.1 ± 5.5	12.1 ± 4.5	9.1 ± 2.5	11.4 ± 4.1	10.1 ± 3.4	−2.2 (0.9–3.5)	−1.7 (0.2–3.2)	−2 (1.1–2.9)	−0.7	**< 0.001**	**< 0.005**

Values are presented as mean ± standard deviation. P-values reflect within-subject comparisons between baseline and post-intervention values, calculated using the Paired T-Test (ESS, ISI, PSQI) and the Wilcoxon Signed Rank Test for non-normally distributed scores (DEPS, GHQ-12). Bonferroni correction for multiple comparisons (m = 5) was applied; both unadjusted and Bonferroni-adjusted p-values (adj p) are reported. Statistical significance after correction was set at adj *p* < 0.05. Effect sizes were estimated using Hedge’s g, corrected for small sample sizes. Abbreviations: ESS, Epworth Sleepiness Scale; ISI, Insomnia Severity Index; PSQI, Pittsburgh Sleep Quality Index; DEPS, Depression Scale; GHQ-12, 12-item General Health Questionnaire

**Table 3:** Changes in symptom parameters in High-Responder subgroup for each key PSG parameter following respiratory muscle training

Symptom Measure (m: w), *n*	Baseline			Post-Intervention			Mean Difference (95% CI)			Total		
	Men	Women	Total	Men	Women	Total	Men	Women	Total	Hedges’ g	*p*	adj *p*
ESS (7:7), *n* = 14	4.1 ± 2.2	9.7 ± 5.0	6.9 ± 4.7	2.6 ± 2.2	6.6 ± 4.6	4.6 ± 4.0	−1.5 (0.2-3)	−3.1 (1.3-5)	−2.3 (1.3–3.4)	−1.2	**< 0.001**	**< 0.005**
ISI (11:6), *n* = 17	10.5 ± 5.4	8 ± 2	9.6 ± 4.5	6.6 ± 5.4	3.5 ± 2.7	5.5 ± 4.8	−3.9 (1.1–6.7)	−4.5 (2.4–6.6)	−4.1 (2.3–5.9)	−1.1	**< 0.001**	**< 0.005**
DEPS (6:7), *n* = 13	5 ± 2.3	4.3 ± 3.4	4.6 ± 2.8	3.2 ± 2.1	2.6 ± 3.8	2.8 ± 3.0	−1.8 (0.4–3.2)	−1.7 (1-2.4)	−1.8 (1.2–2.4)	−1.6	**< 0.001**	**< 0.005**
GHQ-12 (14:10), *n* = 24	24 ± 5.9	21.9 ± 5.2	23.1 ± 5.6	13 ± 6	10.8 ± 6.6	11.8 ± 6.2	−11 (7–15)	−11.1 (7–15)	−11.3 (9–14)	−2	**< 0.001**	**< 0.005**
PSQI (15:7), *n* = 22	10.8 ± 3.4	16 ± 3.2	12.8 ± 4.6	8.3 ± 2.2	12.4 ± 3.2	9.6 ± 3.2	−2.5 (1.6–4.4)	−3.6 (1.2-6)	−3.2 (2.1–4.3)	−1.3	**< 0.001**	**< 0.005**

Values are presented as mean ± standard deviation. P-values in the last column reflect within-subject comparisons between baseline and post-intervention values according to Paired T-Test (ESS, ISI, PSQI) and Wilcoxon Signed Rank Test (DEPS, GHQ-12). Bold text indicates a statistically significant change (*p* < 0.05). Effect sizes were estimated using Hedge’s g, corrected for small sample sizes. P-values for pre- and post-intervention comparisons were obtained using the Paired T-Test for normally distributed variables (ESS, ISI, and PSQI), and the Wilcoxon Signed Rank Test for non-normally distributed variables (DEPS and GHQ-12). M: W = number of men (m) and women (w). N = sample size. Abbreviations: ESS = Epworth Sleepiness Scale, ISI = Insomnia Severity Index, PSQI = Pittsburgh Sleep Quality Index, DEPS = Depression Scale, GHQ-12 = 12-item General Health Questionnaire

**Fig. 2:**
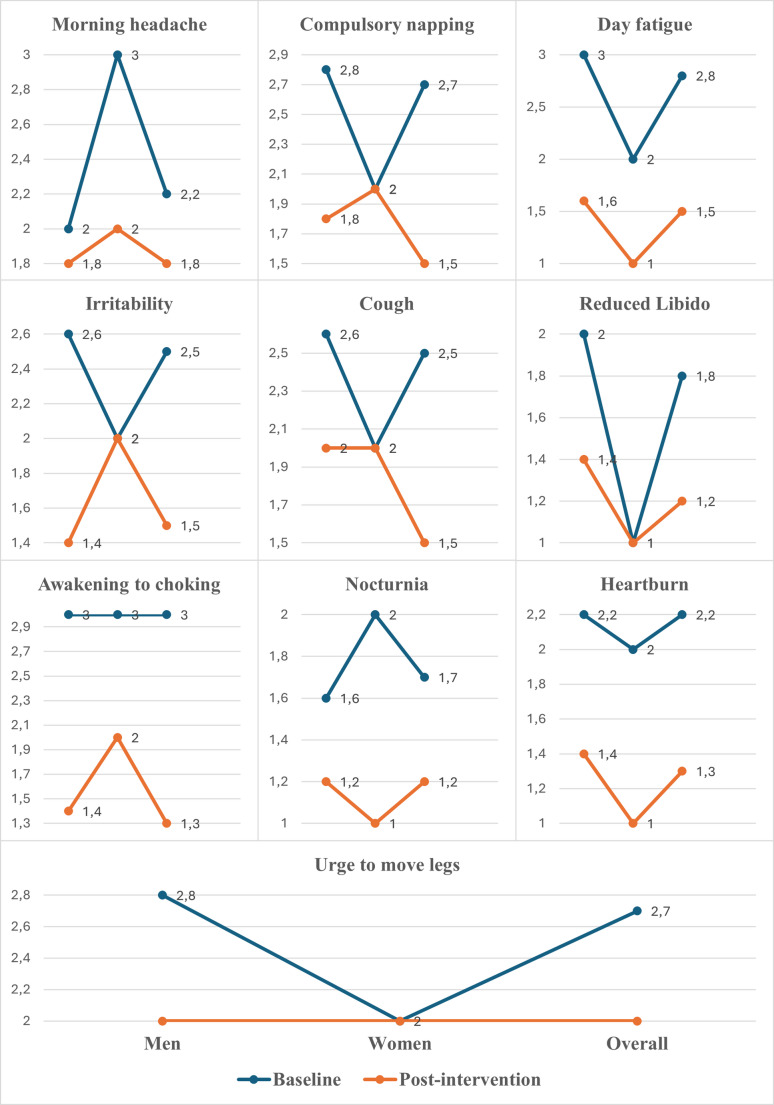
Changes in symptom scores from baseline to post-intervention. Values are presented as mean by gender and overall. Statistical analysis according to Wilcoxon signed rank rest, Bonferroni correction for multiple comparisons (m=19) was applied

In the clinical sleep-apnoea symptom questionnaire, statistically significant reductions in overall symptom frequency after the 12-week RMT programme were observed in morning headache, compulsory daytime napping, daytime fatigue, irritability, cough, reduced libido, episodes of awakening due to choking, nocturnia, heartburn, and urge to move legs (Fig. 2). However, subgroupd analysis by gender revealed that only the reduction in the urge to move legs reached statistical significance among men.

### Pulmonary function tests

A slight improvement in FEV₁ was observed for the overall cohort, with mean values rising from 3.6 L to 3.8 L (*p* = 0.04). FEV₁% of predicted increased from 102.4 to 109.7% (*p* = 0.01) **(**Table 4**)**.

**Table 4:** Changes in pulmonary function parameters in participants following respiratory muscle training

Function Measure	Baseline			Post-Intervention			Mean Difference (95% CI)			Total		
	Men	Women	Total	Men	Women	Total	Men	Women	Total	Hedge’s g	*p*	adj *p*
MIP (cmH₂O)	69.4 ± 24	54.1 ± 26	62.9 ± 26	75.8 ± 27	60 ± 24	69.1 ± 27	6.4 (−0.7, 0.2)	5.9 (−0.7, 0.4)	6.2 (−8, 20)	1.2	0.9	1
MEP (cmH₂O)	79.4 ± 35	55.4 ± 22	69.2 ± 32	73.4 ± 23	60.2 ± 22	67.8 ± 23	−6 (−0.2, 0.7)	4.8 (−0.8, 0.3)	−1.5 (−16,13)	0.2	0.4	1
PEF (L/Min)	621 ± 114	448 ± 61	548 ± 128	637 ± 137*	451 ± 64*	558 ± 146	16 (−0.7, 0.2)	3 (−0.6, 0.5)	10 (−63, 83)	0.9	0.6	1
FVC (L)	5 ± 0.6	3.1 ± 0.7	4.2 ± 1.1	5.1 ± 0.7*	3.1 ± 0.6*	4.24 ± 1.2	0.1 (−0.6, 0.3)	0 (−0.5, 0.6)	0.04 (−1, 1)	−0.08	0.6	1
FVC (%)	102.6 ± 11	88 ± 22	96.4 ± 18	111 ± 15*	81.7 ± 23*	98.8 ± 24	8.4 (−1, −0.06)	−6.3 (−0.3, 0.8)	2.4 (−9, 14)	−0.1	0.5	1
FEV₁ (L)	4.3 ± 0.7	2.8 ± 0.5	3.6 ± 0.9	4.4 ± 0.7*	2.9 ± 0.6*	3.8 ± 1.0	0.1 (−0.9, 0.07)	0.1 (−0.9, 0.2)	0.2 (−0.3, 1)	−0.4	**0.04**	0.4
FEV₁ (%)	109.5 ± 13	92.8 ± 17	102.3 ± 17	117 ± 16*	99.5 ± 19*	110 ± 19	7.5 (−1, −0.07)	6.7 (−0.9, 0.2)	7.3 (−2, 17)	−0.5	**0.01**	0.1
FEV₁(L)/FVC(L)	89.2 ± 7	92.9 ± 7	90.8 ± 7	88.3 ± 7*	93.6 ± 7*	90.5 ± 7	−0.9 (−0.3, 0.6)	1.7 (−0.7, 0.4)	−0.2 (−4, 3)	−0.2	0.8	1
FEV₁(%)/FVC(%)	112.3 ± 8	117.7 ± 9	114.6 ± 9	113 ± 9*	118.2 ± 9*	115 ± 9.1	0.7 (−0.5, 0.4)	0.5 (−0.6, 0.5)	0.4 (−4, 5)	0.2	0.2	1

Values are presented as mean ± standard deviation. P-values reflect within-subject comparisons between baseline and post-intervention values, calculated using paired t-tests for normally distributed variables (MIP, PEF, FVC, FEV₁, FEV₁/FVC ratio), and the Wilcoxon signed rank test for non-normally distributed variables (MEP). Bonferroni correction was applied for multiple comparisons (m = 9); both unadjusted (p) and Bonferroni-adjusted (adj p) p-values are reported, with statistical significance set at adj *p* < 0.05. Effect sizes were calculated using Hedge’s g, corrected for small sample sizes. Abbreviations: MIP, Maximal Inspiratory Pressure (cmH₂O); MEP, Maximal Expiratory Pressure (cmH₂O); PEF, Peak Expiratory Flow (L/min); FVC, Forced Vital Capacity (L and % of predicted); FEV₁, Forced Expiratory Volume in one second (L and % of predicted)

Moderate variability of changes was noted in FVC, MIP, MEP, and PEF, each displaying individual improvement in some participants but no uniform change across the entire group was found. Among the subgroup demonstrating positive outcomes, Table 5 highlights substantial gains across both inspiratory and expiratory measures.

**Table 5:** Changes in pulmonary function parameters in High-Responder subgroups following respiratory muscle training

Function Measure (m: w), *n*	Baseline			Post-Intervention			Mean Difference			Total		
	Men	Women	Total	Men	Women	Total	Men	Women	Total	Hedges’ g	*p*	adj *p*
MIP (cmH₂O) (11:8), *n* = 19	66.6 ± 25	43.3 ± 20.6	57.7 ± 25.9	86.9 ± 26	72 ± 20.5	82.8 ± 23.1	20.3	28.7	25.1	−1.1	< 0.001	< 0.009
MEP (cmH₂O) (6:8), *n* = 14	64.2 ± 24.7	46.6 ± 21.2	54.1 ± 23.6	76.8 ± 19	63.3 ± 22.9	69.1 ± 21.5	12.6	16.7	14.9	−1	0.004	0.04
PEF (L/Min) (9:10), *n* = 19	639 ± 121	439 ± 38	535 ± 133	711 ± 99	468 ± 38	584 ± 142	72	29	48.8	−1	< 0.001	< 0.009
FVC (L) (13:5), *n* = 18	5 ± 0.8	2.8 ± 0.3	4.36 ± 1.5	5.3 ± 0.8	3.3 ± 0.3	4.68 ± 1.1	0.3	0.5	0.3	−1.5	< 0.001	< 0.009
FVC (%) (16:4), *n* = 20	102 ± 9.8	76.8 ± 10.3	96.4 ± 14.2	114 ± 14	94.3 ± 17.6	110.7 ± 16.4	12	17.5	14.4	−1.1	< 0.001	< 0.009
FEV₁ (L) (12:7), *n* = 19	4.3 ± 0.5	2.9 ± 0.5	3.8 ± 0.8	4.6 ± 0.6	3.3 ± 0.4	4.1 ± 0.9	0.3	0.4	0.3	−0.9	< 0.001	< 0.009
FEV₁ (%) (13:8), *n* = 21	106.7 ± 11	83.6 ± 12.8	97.9 ± 16	121 ± 15	102 ± 17.8	113.7 ± 18.3	14.3	18.4	15.8	−1.3	< 0.001	< 0.009
FEV₁(L)/FVC(L) (6:7), *n* = 13	85 ± 7.2	88.9 ± 7.2	87.1 ± 7.2	92 ± 4.5	92.7 ± 6.9	92.5 ± 5.7	7	3.8	5.5	−1.2	< 0.001	< 0.009
FEV₁(%)/FVC(%) (8:9), *n* = 17	110 ± 9	113 ± 10	112.2 ± 9.1	118 ± 7.4	120 ± 9.2	119 ± 8	8	7	6.9	−1.3	< 0.001	< 0.009

Values are presented as mean ± standard deviation. P-values reflect within-subject comparisons between baseline and post-intervention values, calculated using paired t-tests for normally distributed variables (MIP, PEF, FVC, FEV₁, FEV₁/FVC ratio) and the Wilcoxon signed rank test for non-normally distributed variables ((MEP). Bonferroni correction was applied for multiple comparisons (m = 9); both unadjusted (p) and Bonferroni-adjusted (adj p) p-values are reported, with statistical significance set at adj *p* < 0.05. Effect sizes were calculated using Hedge’s g, corrected for small sample sizes. M: W = number of men (m) and women (w). N = sample size. Abbreviations: MIP, Maximal Inspiratory Pressure (cmH₂O); MEP, Maximal Expiratory Pressure (cmH₂O); PEF, Peak Expiratory Flow (L/min); FVC, Forced Vital Capacity (L and % of predicted); FEV₁, Forced Expiratory Volume in one second (L and % of predicted)

Abbreviations: AHI = apnoea-hypopnoea index

### Determinants

The potential influence of individual characteristics including gender, age, height, weight, waist circumference, neck circumference, multimorbidity, and smoking status, on changes in outcome variables (ISI, GHQ-12, PSQI, FEV₁ [L], and FEV₁ [%]) was assessed using linear regression analysis. None of the independent variables, including central obesity, gender, anthropometric measures, smoking, or comorbidity status, predicted changes in these outcome variabes.

Logistic sensitivy analyses using dichotomized literature-based thresholds found no independent associations of central obesity or composite comorbidity with meeting symptom cut-offs. A nominally protective association for comorbidity with ESS (OR 0.11, 95% CI 0.01–0.9) is based on six events and should be considered unstable. The ≥ 5 events-per-variable (EPV) rule was maintained in all models to ensure minimum statistical reliability.

## Discussion

This open-label pilot study of prospective clinical intervention of steam-assisted respiratory muscle training for 12 weeks suggests the intervention may improve quality of life and alleviated symptoms in adults with mild to moderate obstructive sleep apnoea. However, these findings must be interpreted with caution due to the study’s open-label design, lack of a control group, and modest sample size.

The results show reductions in ISI, GHQ-12, and PSQI scores indicate that steam-assisted training can alleviate core OSA symptoms like insomnia and psychological distress, even if daytime sleepiness (ESS) and depressive symptoms (DEPS) were unchanged. Nonetheless, an especially responsive subgroups showed improvements in virtually all subjective measures. These findings are in line with a previous study were five weeks’ RMT improved quality of life without improvements in AHI, pulmonary function or daytime sleepiness [8]. While severe OSA is consistently associated with cardiovascular and metabolic risks, the effects of mild to moderate OSA remain less clear, partly due to limitations in standard definitions and small sample sizes in past studies [23].

Marked gender-based differences emerged in anthropometric parameters, as women generally presented higher BMI and waist circumference, while men had a greater smoking prevalence, both recognized risk factors for OSA progression [8]. This observation aligns with previous studies indicating potential gender-based differences in OSA risk factors and symptom severity [24]. However, while the results highlight differences in certain anthropometrics and lifestyle factors, it remains unclear whether these variations substantially affect RMT efficacy. Some earlier investigations have examined gender differences in OSA interventions, but findings remain inconclusive regarding whether men and women respond differently to respiratory muscle training or other therapies [9, 25]. The balanced enrollment of men and women provides a foundation for future analyses aimed at clarifying whether gender-specific factors modulate RMT outcomes in mild to moderate OSA. Comorbidities were present in 76% of participants, primarily cardiovascular and metabolic disorders, typical ones in OSA populations. This prevalence appears notably higher than in the general population, where the burden of cardiometabolic conditions is lower among individuals without OSA [25]. While several statistically significant differences were observed, excluded participants had higher BMI, AHI, and PSQI scores, and lower respiratory function values (all of which could be related to the exclusion criteria of severe OSA), no systematic bias was evident beyond these measures. These findings suggest that, despite these differences, the final cohort remains broadly representative of real-world patients with mild to moderate OSA.

Responses to the clinical sleep-apnoea questionnaire showed broad improvement after the 12-week steam-assisted RMT. Statistically significant reductions in overall symptom frequency were observed for morning headache (Fig. 2), compulsory daytime napping, daytime fatigue, irritability, cough, reduced libido, episodes of awakening due to choking, nocturia, heartburn, and urge to move legs. Within the restless-leg domain, median scores for “urge to move legs,” “symptoms worse at rest,” and “relief after walking” each shifted downward by one category with narrower inter-quartile ranges, indicating both a left-shift and reduced dispersion.

In gender-stratified analyses, only the reduction in the urge to move legs reached statistical significance among men; other within-gender contrasts did not reach significance, consistent with limited subgroup power. Taken together, these distribution-based results confirm that targeted respiratory-muscle training can attenuate a broad spectrum of OSA-related complaints, while also highlighting that the pattern of benefit is not identical in men and women. Literature on gender differences in RMT effects remains limited; most clinical trials have been small and predominantly male, limiting the power to detect gender-specific effects; a small study by Verma et al. [26] examining oropharyngeal exercises suggests potential differences in therapeutic responses, though the female sample was too small for definitive conclusions. Women commonly present distinct symptom profiles, such as higher prevalence of morning headaches and mood disturbances, whereas men frequently report loud snoring [24]. Currently, it is not well known if RMT benefits men and women equally; future studies should specifically explore gender-related responses to clarify potential disparities.

The improvement in FEV₁ supports the hypothesis that fortifying respiratory musculature can boost upper airway stability, thereby mitigating apneic events [25, 27, 28]. In contrast, FVC, MIP, MEP, and PEF exhibited variability at the group level, though a subset of “high responders” registered robust gains in these parameters. Such heterogeneity underscores the multifactorial nature of OSA and highlights the importance of personalized approaches, including adherence and baseline physiological capacity [5].

While CPAP remains the first-line therapy for moderate to severe OSA, its long-term effectiveness is often limited by adherence challenges [4, 5]. This study suggests steam-assisted RMT as a non-invasive alternative that enhances pulmonary function and subjective well-being. Unlike CPAP, which maintains airway patency through positive pressure, RMT may strengthen upper airway musculature, potentially yielding lasting functional benefits.

Key strengths of this study include its evaluation of both pulmonary function and subjective, symptom-related outcomes. The inclusion of both male and female participants allowed for an analysis of potential differences in response to the intervention between genders. Furthermore, the study utilized the WellO2^®^ device, which combines both inspiratory and expiratory resistance with heated steam, an approach that distinguishes it from many prior RMT studies. The study also involved the analysis of subgroups to identify participants who demonstrated more pronounced improvements in validated questionnaires [27, 28].

The study has some limitations. Most importantly, because no control arm was included, observed changes may partly reflect regression to the mean, placebo effects, or selection bias rather than a true intervention effect. A control arm was not included because creating a sham device that would be indistinguishable to participants was not practically or ethically feasible for this device (credible shams for non-pharmacologic/device interventions are often difficult to design and maintain, and may expose participants to risks without prospect of direct benefit). An elevated dropout rate diminished study’s final sample size beneath the initial objective. A distinct subgroup of dropouts had severe OSA measured with AHI in the first PSG. This is attributable in part to discrepancies between the home respiratory polygraphy (PG) utilized at referral and the in-lab polysomnography conducted, as PSG-derived AHI values may be up to 30% greater [29]. Moreover, two distinct overnight recordings may produce significantly divergent outcomes [30]. Secondly, we lacked an objective metric for WellO2^®^ device utilization, rendering it uncertain whether all participants employed the device as prescribed. Furthermore, the questionnaires used did not allow for a sub-analysis of insomnia subtypes, such as difficulties with sleep onset versus sleep maintenance, which would have offered deeper insight into the intervention’s specific effects. Finally, the study’s criteria, including the exclusion of patients with AHI > 30/h, limit the generalizability to individuals with severe OSA.

## Conclusion

In this pilot study, 12 weeks of steam-assisted respiratory muscle training was associated with enhanced mental health, as demonstrated by reduced GHQ-12 scores, implying a more extensive impact on well-being. While larger trials are needed to confirm these preliminary findings, these pilot results do not yet demonstrate sustained benefit and should be interpreted with caution. In an era of highlighting personalized care, non-invasive therapeutic devices like WellO2^®^ may emerge as viable alternatives for add-on management of sleep-disordered breathing.

## Supplementary Information

Below is the link to the electronic supplementary material.

Supplementary Material 1

Supplementary Material 2

## Data Availability

De-identified data that support the findings are available from the corresponding author upon reasonable request, subject to institutional ethics approval.

## References

[1] Peppard PE, Young T, Palta M, Skatrud J (2000) Prospective study of the association between Sleep-Disordered breathing and hypertension. N Engl J Med 342(19):1378–1384. 10.1056/NEJM200005113421901 10805822 10.1056/NEJM200005113421901

[2] Vanderveken OM, Boudewyns A, Ni Q et al (2011) Cardiovascular implications in the treatment of obstructive sleep apnea. J Cardiovasc Trans Res 4:53–60. 10.1007/s12265-010-9238-y 10.1007/s12265-010-9238-y 21104045

[3] Jordan AS, McSharry DG, Malhotra A (2014) Adult obstructive sleep Apnoea. Lancet 383(9918):736–747. 10.1016/S0140-6736(13)60734-5 23910433 10.1016/S0140-6736(13)60734-5 PMC3909558

[4] Afsharpaiman S, Shahverdi E, Vahedi E, Aqaee H (2016) Continuous positive airway pressure compliance in patients with obstructive sleep apnea. Tanaffos 15(1):25–30 27403175 PMC4937758

[5] Turino C, Benítez ID, Rafael-Palou X, Mayoral A, Lopera A, Pascual L, Vaca R, Cortijo A, Moncusí-Moix A, Dalmases M, Vargiu E, Blanco J, Barbé F, de Batlle J (2021) Management and treatment of patients with obstructive sleep apnea using an intelligent monitoring system based on machine learning aiming to improve continuous positive airway pressure treatment compliance: randomized controlled trial. J Med Internet Res 23(10):e24072. 10.2196/24072 34661550 10.2196/24072 PMC8561405

[6] Dempsey JA, Veasey SC, Morgan BJ, O’Donnell CP (2010) Pathophysiology of sleep apnea. Physiol Rev. ;90(1):47–112. 10.1152/physrev.00043.2008. Erratum in: Physiol Rev.2010;90(2):797-8 10.1152/physrev.00043.2008 PMC3970937 20086074

[7] Eckert DJ (2018) Phenotypic approaches to obstructive sleep apnoea - New pathways for targeted therapy. Sleep Med Rev 37:45–59. 10.1016/j.smrv.2016.12.003Epub 2016 Dec 18. PMID: 28110857 28110857 10.1016/j.smrv.2016.12.003

[8] Herkenrath SD, Treml M, Priegnitz C, Galetke W, Randerath WJ (2018) Effects of respiratory muscle training (RMT) in patients with mild to moderate obstructive sleep apnea (OSA). Sleep Breath 22(2):323–328. 10.1007/s11325-017-1582-6 29080065 10.1007/s11325-017-1582-6

[9] Hsu B, Emperumal CP, Grbach VX, Padilla M, Enciso R (2020) Effects of respiratory muscle therapy on obstructive sleep apnea: a systematic review and meta-analysis. J Clin Sleep Med 16(5):785–801. 10.5664/jcsm.8318 32026802 10.5664/jcsm.8318 PMC7849810

[10] Kuronen I, Heinijoki J, Sovijärvi A (2024) Effects of low workload respiratory training with steam inhalation on lung function in stable asthma: A controlled clinical study. Clin Physiol Funct Imaging 44(1):100–111. 10.1111/cpf.12856 37749950 10.1111/cpf.12856

[11] Camacho M, Certal V, Abdullatif J, Zaghi S, Ruoff CM, Capasso R, Kushida CA (2015) Myofunctional therapy to treat obstructive sleep apnea: A systematic review and meta-analysis. Sleep (New York, N.Y.) 38(5):675. 10.5665/sleep.4652 10.5665/sleep.4652 PMC4402674 25348130

[12] Dar JA, Mujaddadi A, Moiz JA (2022) Effects of inspiratory muscle training in patients with obstructive sleep Apnoea syndrome: A systematic review and meta-analysis. Sleep Sci (São Paulo SP) 15(4):480–489. 10.5935/1984-0063.20220081 10.5935/1984-0063.20220081 PMC9670769 36419804

[13] American Thoracic Society/European Respiratory Society. ATS/ERS Statement on respiratory muscle testing. Am J Respir Crit Care Med (2002) ;166(4):518–624. 10.1164/rccm.166.4.518. PMID: 12186831 10.1164/rccm.166.4.518 12186831

[14] Miller MR, Hankinson J, Brusasco V, Burgos F, Casaburi R, Coates A, Crapo R, Enright P, van der Grinten CP, Gustafsson P, Jensen R, Johnson DC, MacIntyre N, McKay R, Navajas D, Pedersen OF, Pellegrino R, Viegi G, Wanger J (2005) ATS/ERS task force. Standardisation of spirometry. Eur Respir J 26(2):319–338. 10.1183/09031936.05.00034805 16055882 10.1183/09031936.05.00034805

[15] Johns MW (1991) A new method for measuring daytime sleepiness: the Epworth sleepiness scale. Sleep 14(6):540–5. 10.1093/sleep/14.6.540 1798888 10.1093/sleep/14.6.540

[16] Buysse DJ, Reynolds CF 3rd, Monk TH, Berman SR, Kupfer DJ (1989) The Pittsburgh Sleep Quality Index: a new instrument for psychiatric practice and research. Psychiatry Res 28(2):193–213. 10.1016/0165-1781(89)90047-4 2748771 10.1016/0165-1781(89)90047-4

[17] Morin CM (1993) Insomnia: psychological assessment and management. Guilford Press, New York

[18] Bastien CH, Vallières A, Morin CM (2001) Validation of the insomnia severity index as an outcome measure for insomnia research. Sleep Med 2(4):297–307. 10.1016/s1389-9457(00)00065-4 11438246 10.1016/s1389-9457(00)00065-4

[19] Salokangas RK, Poutanen O, Stengård E (1995) Screening for depression in primary care. Development and validation of the depression scale, a screening instrument for depression. Acta Psychiatr Scand 92(1):10–16. 10.1111/j.1600-0447.1995.tb09536.x 7572242 10.1111/j.1600-0447.1995.tb09536.x

[20] Goldberg DP, Williams P (1988) A user’s guide to the General Health Questionnaire. NFER-Nelson

[21] World Health Organization (‎2011)‎. Waist circumference and waist-hip ratio: report of a WHO expert consultation, Geneva, 8–11 December 2008. World Health Organization. https://iris.who.int/handle/10665/44583

[22] Peduzzi P, Concato J, Kemper E, Holford TR, Feinstein AR (1996) A simulation study of the number of events per variable in logistic regression analysis. J Clin Epidemiol 49(12):1373–1379. 10.1016/s0895-4356(96)00236-3 8970487 10.1016/s0895-4356(96)00236-3

[23] Peppard PE, Hagen EW (2018) The last 25 years of obstructive sleep apnea Epidemiology-and the next 25? Am J Respir Crit Care Med 197(3):310–312. 10.1164/rccm.201708-1614PP 29035088 10.1164/rccm.201708-1614PP

[24] Saaresranta T, Anttalainen U, Polo O (2015) Sleep disordered breathing: is it different for females? ERJ Open Res 1(2):00063–2015. 10.1183/23120541.00063-2015 27730159 10.1183/23120541.00063-2015 PMC5005124

[25] Borges LS, Muniz MT, Fregonezi G (2022) Inspiratory muscle training in obstructive sleep apnea: A systematic review and meta-analysis. Nat Sci Sleep 14:1999–2016

[26] Verma RK, Johnson J Jr, Goyal M, Banumathy N, Goswami U, Panda NK (2016) Oropharyngeal exercises in the treatment of obstructive sleep apnoea: our experience. Sleep Breath 20:1193–1201. 10.1007/s11325-016-1332-1 26993338 10.1007/s11325-016-1332-1

[27] Silva de Sousa A, Pereira da Rocha A, Brandão Tavares DR, Frazão Okazaki JÉ, de Andrade Santana MV (2024) Fernandes Moça Trevisani V, Pereira Nunes Pinto AC. Respiratory muscle training for obstructive sleep apnea: systematic review and meta-analysis. J Sleep Res 33(3):e13941. 10.1111/jsr.13941 37258418 10.1111/jsr.13941

[28] Azeredo LM, Souza LC, Guimarães BLS, Puga FP, Behrens NSCS, Lugon JR (2022) Inspiratory muscle training as adjuvant therapy in obstructive sleep apnea: A randomized controlled trial. Braz J Med Biol Res 55:e12331. 10.1590/1414-431X2022e12331 36197415 10.1590/1414-431X2022e12331 PMC9529044

[29] Escourrou P, Grote L, Penzel T, Mcnicholas WT, Verbraecken J, Tkacova R, Riha RL, Hedner J, ESADA Study Group (2015) The diagnostic method has a strong influence on classification of obstructive sleep apnea. J Sleep Res 24(6):730–738. 10.1111/jsr.12318 26511017 10.1111/jsr.12318

[30] Lechat B, Naik G, Reynolds A, Aishah A, Scott H, Loffler KA, Vakulin A, Escourrou P, McEvoy RD, Adams RJ, Catcheside PG, Eckert DJ (2022) Multinight prevalence, variability, and diagnostic misclassification of obstructive sleep apnea. Am J Respir Crit Care Med 205(5):563–569. 10.1164/rccm.202107-1761OC 34904935 10.1164/rccm.202107-1761OC PMC8906484

