# Supplementary material for: Effects of steam-assisted respiratory muscle training on sleep apnoea symptoms and pulmonary function in men and women: a pilot study
Source: Sleep Breath. 2025 Sep 15;29(5):286. doi: 10.1007/s11325-025-03449-2 (PMC12436549; doi:10.1007/s11325-025-03449-2)
Supplement: Supplementary file 1 — Supplementary Material 1 [file 11325_2025_3449_MOESM1_ESM.docx]

**Supplement 1. Distribution of comorbidities among study participants.** The horizontal bar chart displays the number of participants reporting each comorbid condition, grouped by clinical category. Comorbidity categories are based on affected organ systems or underlying pathophysiological mechanisms and include cardiovascular, gastrointestinal, genitourinary, immunological, metabolic, neurological, and other. Color coding corresponds to comorbidity classification. Notably, hypertension, hyperlipidemia, and connective tissue disease were the most reported comorbidities in the study cohort.
