# Supplementary material for: Effects of steam-assisted respiratory muscle training on sleep apnoea symptoms and pulmonary function in men and women: a pilot study
Source: Sleep Breath. 2025 Sep 15;29(5):286. doi: 10.1007/s11325-025-03449-2 (PMC12436549; doi:10.1007/s11325-025-03449-2)
Supplement: Supplementary file 2 — Supplementary Material 2 [file 11325_2025_3449_MOESM2_ESM.docx]

**Supplement 2: Distribution of prescribed medicines categorized by Anatomical Therapeutic Chemical (ATC) according to the WHO classification system.** Each bar represents the number of medications prescribed within each ATC code category. The most frequently represented groups included lipid-modifying agents (C10), psychoanaleptics (N06), and agents acting on the renin-angiotensin system (C09).

ATC codes represented in the data include: A02 (acid related disorders), A03 (functional gastrointestinal disorders), A07 (antidiarrheals, intestinal anti-inflammatory/anti-infective agents), A10 (drugs used in diabetes), A11 (vitamins), A12 (mineral supplements), B01 (antithrombotic agents), B03 (antianemic preparations), C01 (cardiac therapy), C02 (antihypertensives), C07 (beta blocking agents), C08 (calcium channel blockers), C09 (agents acting on the renin-angiotensin system), C10 (lipid modifying agents), D01 (antifungals for dermatological use), G03 (sex hormones and modulators of the genital system), G04 (urologicals), H02 (corticosteroids for systemic use), H03 (thyroid therapy), L01 (antineoplastic agents), L04 (immunosuppressants), M01 (anti-inflammatory and antirheumatic products), N02 (analgesics), N05 (psycholeptics), N06 (psychoanaleptics), R01 (nasal preparations), R03 (drugs for obstructive airway diseases), and R06 (antihistamines for systemic use).
